# Parenting Practices in Pregnancy Smokers Compared to Non Smokers

**DOI:** 10.4021/jocmr1283w

**Published:** 2013-02-25

**Authors:** Mini Tandon, Xuemei Si, Andy Belden, Ed Spitznagel, Lauren S. Wakschlag, Joan Luby

**Affiliations:** a Department of Psychiatry, Washington University School of Medicine (WUSM), 660 South Euclid Avenue, St. Louis, MO, USA; b GTx, Inc., 175 Toyota Plaza #700, Memphis, TN 38103, USA; c Department of Mathematics, Washington University in St. Louis, One Brookings Drive, St. Louis, MO 63130, USA; d Department of Medical Social Sciences, Northwestern University Feinberg School of Medicine, 420 East Superior, Street, Chicago, Il 60611, USA

**Keywords:** Prenatal, Smoking, Disruptive behavior, Parenting

## Abstract

**Background:**

The present investigation compared parenting practices in a sample of preschoolers whose mothers reported smoking during pregnancy versus those who did not.

**Methods:**

A sample of n = 216, 3.0- to 5.11-year-old children, participants in an ongoing longitudinal study, was separated into those reportedly exposed to smoking in utero and those who were not. Parenting practices were compared between the two groups, using T-tests and exact logistic regressions. Multiple linear regressions and multivariate logistic regressions were used to examine the association between smoking status and parenting, controlling for variables also known to be associated with parenting practices.

**Results:**

Current study findings suggest that smoking during pregnancy is associated with harsh parenting practices.

**Conclusions:**

Study results highlight the possible role of parenting in disruptive outcomes well-known in toddlers exposed to nicotine in utero and have implications for targeting early interventions in these populations.

## Introduction

An association between prenatal cigarette exposure and disruptive behavior in childhood has been reported in the empirical literature from multiple independent investigations [1-3]. However, whether parent-child relationship factors and other psychosocial factors may also be important in this risk trajectory has not been adequately addressed in the literature. The potential importance of early parent-child relational risks in this domain is underscored by studies that suggest that maternal responsiveness can moderate the effect of prenatal cigarette exposure on disruptive outcomes in preschoolers [4], and that parental responsiveness has moderated disruptive behavior as late as adolescence [5]. While parenting of preschoolers seems a modifiable target for intervention to reduce disruptive outcomes, it has not been a key area of focus for mothers who continue to smoke cigarettes through pregnancy. To our knowledge, despite studies of this issue in school age children, this is the first study to examine specific parenting practices in mothers of preschoolers who smoked during pregnancy. Further, the importance of earlier interventions in the treatment of disruptive behavior has been well established [6-9].

### Examining risk factors for smoking while pregnant

Numerous individual and psychosocial differences have been found between mothers that smoke during pregnancy and mothers that do not. Such differences have included the findings of more antisocial character traits in smoking mothers and a higher likelihood of selecting mates with these traits [10]. Mothers who smoke while pregnant have been found to come from more adverse social circumstances and economically disadvantaged backgrounds [10, 11]. They are often younger than non-smoking mothers [12]. Women who smoke while pregnant have been found to have difficulty with mood regulation, presumptively using cigarettes as a coping mechanism, as smoking cigarettes to regulate mood has been an empirically established phenomenon [13-16]. Pregnancy smokers tend to be more impulsive, with a history of externalizing problems, when compared to mothers that quit smoking while pregnant [17], and increased impulsivity has been found among pregnancy smokers compared to non-smokers [18]. Furthermore, decreased maternal warmth and increased hostility have also been demonstrated during early infancy among smoking mothers [19, 20]. Other psychosocial risk factors pertinent to parenting such as poor adaptive functioning and problems in interpersonal relationships have been shown to incrementally predict pregnancy smoking status after controlling for demographic risks [21].

The above mentioned personal and psychosocial features of pregnancy smokers may themselves contribute to poor parenting skills. To further complicate the challenges of parenting in this population, the child of a pregnancy smoker may also be more difficult to parent than the child of a non-smoker on the basis of biological risk factors. For example, while the mechanism remains unclear, smoking while pregnant has been associated with increased negativity in toddlers measured as rebelliousness, risk taking, and impulsivity, behaviors often found in children with disruptive disorders [22]. Brook et al [22] found that after controlling for psychosocial risks (such as demographics, parental intrapersonal difficulty, and parenting difficulties) commonly associated with early childhood problem behaviors, the relationship between smoking during pregnancy and child’s negativity remained significant. Taken together, these findings suggest that not only is smoking during pregnancy associated with key risk factors in the mother, but it also independently confers a risk to the child for which enhanced parenting skills are needed. While some differences which contribute to risk for smoking while pregnant are not easily modifiable, others, such as parental responsiveness and parental discipline, might prove to be feasible targets for early intervention efforts [5].

### Smoking and parenting

To better understand the relationship between smoking in pregnancy and early child behavioral problems, further examination of parenting among pregnancy smokers compared to non-smokers is now warranted. There is a dearth of published studies that specifically inform this issue. While most prior investigations have focused on prenatal smoking and risk for disruptive behavior outcomes in the child, parenting constructs were also examined in some of these studies. These available study findings have suggested that mothers who smoked showed less parental nurturing, as well as poor behavioral management and dyadic communication skills [4, 23, 24]. Specifically, Wakschlag et al [23] examined communication, discipline, and supervision at initial assessment as factors thought to be related to the pathway of conduct disorder when male participants were between the ages 7 and 12. Similarly, parents of children with a mean age of 16.4 years old were queried regarding parent-child discord, affectionless control, and cohesiveness in a sample of n = 147 child-mother pairs with prenatal smoking exposure [24]. While both studies examined parenting as part of an investigation on prenatal cigarette exposure and included development of disruptive psychopathology, the participants were older children. On the other end of the age spectrum, parenting was examined during infancy at 4, 12, and 24 months of age, using observational measures of maternal responsiveness in a sample of n = 77 10-year-old children [4]. While this study found that early maternal responsiveness plays a protective role in the risk trajectory of prenatal smoking exposure to child disruptive behavior, it did not examine parenting measures specific to the preschool period. Similarly, Wakschlag et al [3] examined parenting and early emergence of disruption in n = 95 prenatally exposed infants through age 24 months.

Additional studies have examined parenting among pregnancy smokers at multiple stages of development, from neonates [20] to adolescents [5]; however, few investigations have focused on the preschool period. Whether developmental effects can be identified would be useful to inform periods of lower or higher risk to target intervention and/or prevention. The current study aimed to investigate what differences, if any, existed in parenting in a sample of preschoolers whose mothers smoked (“pregnancy smokers”) versus did not during pregnancy. The preschool period was thought to be particularly important given the central role of parenting to the child’s development at this stage. To our knowledge, this is the first study to focus on parenting practices specific to the preschool period in children prenatally exposed to cigarettes. The current study focused on problematic parenting behaviors to inform potential targets for intervention in the risk pathway from prenatal cigarette exposure to child disruptive outcomes. It was thought that examining specific negative parenting behaviors would shed light on what facets of parenting to address among pregnancy smokers to reduce child disruptive outcomes.

## Methods

### Participants

Preschool age children (n = 306) between the ages of 3.0 and 5.11 and their caregivers were recruited from pediatricians’ offices, daycares, and preschools in the St. Louis metropolitan area using the Preschool Feelings Checklist (PFC) [25], a brief validated screening tool for early-onset emotional disorders for participation in a study of preschool onset depression [26]. Consent was obtained from caregivers in concordance with the WUSM IRB. Children who agreed to participate in the study underwent a comprehensive developmental and mental health assessment. During a 3-hour laboratory visit mothers were interviewed about their preschoolers’ moods and behaviors with a comprehensive semi-structured diagnostic interview that also ascertained information about parenting and smoking during pregnancy. From a total sample of n = 306, n = 216 participants had data available on the presence or absence of prenatal cigarette exposure (n = 177 non-smokers, n = 29 smoked less than 10 cigarettes a day, n = 10 smoked 10 or more cigarettes a day) along with parenting variables and were therefore included in the investigation that follows.

### Measures

#### Parenting: Preschool Age Psychiatric Assessment (PAPA)

The PAPA is an interviewer-based diagnostic assessment with established test-retest reliability designed for use in children ages 3 to 6 [27]. The PAPA includes a Family Section, which assesses the frequency and intensity of parental discipline, verbal dispraise, rejection, and selective negative view. Specifically, parental discipline inquires about use of timeout, spanking with or without an implement, leaving marks and/or bruises, and loss of privileges. Verbal dispraise is described as demeaning the child or “condemnation of a child his/herself instead of his/her actions” (for example, saying “You’re a bad boy” for pulling the cat’s tail instead of “It’s bad to hurt the cat”). Further verbal rejection is defined as “the parent addresses the child with words or a tone that pushes the child away or puts a barrier between them” ( for example, “Does the child ever make you so mad that you say you wish s/he had never been born?”). Finally selective negative view involves a discrepant treatment of one child compared to his/her siblings for equivalent misdemeanors (for example, “Do you find yourself treating him/her differently than the other children for the same misbehavior?”). The PAPA queries information for the last 3 months including symptom onset, duration, frequency, and setting, requiring the interviewer to make certain that the parent has understood the question using examples. Interviewers are trained for 5 days plus 2 - 3 practice interviews and have at least a bachelor’s degree [27]. The PAPA also assesses diagnostic categories according to the DSM-IV and has demonstrated diagnostic reliability (k = 0.36 to 0.79). Test-retest intra-class correlations for DSM-IV syndrome scale scores have also been published (0.56 to 0.89). No age, sex, or race reliability differences have been found [27]. Test-retest reliability of the PAPA is similar to that of established measures for older children, such as the Diagnostic Interview Schedule for Children [28].

#### Smoking exposure: Child and Adolescent Psychiatric Assessment (CAPA)

The CAPA is an interviewer-based, structured psychiatric interview for children with collection of data on symptom onset, frequency, intensity and duration according to DSM-IV, III-R or ICD-10 criteria. Modules such as substance use (tobacco), can be used separately, and test-retest reliability for diagnoses range from k = 0.55 (conduct disorder) to k = 1.0 (substance abuse) [29]. Mothers were queried regarding pregnancy smoking using the CAPA substance use module when children attended their annual assessments for the original longitudinal study.

#### Disruptive and externalizing behavior: Health and Behavior Questionnaire-Parent (HBQ-P)

The HBQ-P is a 140-item reliable and valid parent informant measure that produces dimensional ratings of 4- to 8-year-old children’s functioning in the domains of emotional and behavioral symptomotology, impairment, adaptive social functioning, and physical health using a 3-point Likert scale with a 20-minute completion time [30].

### Procedures

Parenting practices based on self-report were examined in mothers who smoked versus those who did not smoke cigarettes during pregnancy in a sample of n = 306 children enrolled in an ongoing longitudinal investigation of preschoolers. T-tests and exact logistic regressions were used to compare the differences for parenting variables between mothers who smoked and those who did not. Mann-Whitney U tests were used to compare two smoking categories. Multiple linear regressions and multivariate logistic regressions were used to examine the association between smoking status and parenting variables (frequency or intensity) controlling for confounding variables.

## Results

Of the n = 306 preschoolers from the longitudinal investigation, n = 216 participants had data available on both parenting and smoking exposure. Demographic characteristics of study participants included in this analysis are shown in Table 1. Differences between included and excluded participants were examined, and the only difference found was that the excluded group was younger. Differences between study participants (n = 216) included that non-smoking mothers had more college and graduate school level education compared to pregnancy smokers (P < 0.001). Further, non-smoking mothers versus pregnancy smokers tended to report higher income, more specifically in the $60,000 or more range (P = 0.001). Finally, the group of pregnancy smokers consisted of more African American mothers and less Caucasian mothers than the group of non smoking mothers (P = 0.017).

**Table 1:** Demographic Characteristics of the Study Sample (n = 216)

Characteristic	Bio-mom did not-smoke n (%)	Bio-mom Smoked n (%)	χ^2^	P
No. of participants	177 (82)	39 (18)		
Baseline Age, year			0.797	0.671
3	42 (24)	11 (28)		
4	86 (49)	16 (41)		
5	48 (27)	12 (31)
Child Ethnicity			8.187	0.017
Caucasian	107 (61)	14 (36)		
African American	52 (29)	18 (46)		
Other	17 (10)	7 (18)
Child Gender			0.476	0.595
Males	97 (55)	19 (49)		
Females	80 (45)	20 (51)
Total family income at baseline			15.939	0.001
≤ $20,000	26 (16)	16 (46)		
$20,001 - $40,000	30 (18)	4 (11)		
$40,001 - $60,000	32 (19)	6 (17)		
≥ $60,001	76 (46)	9 (26)		
Bio-mom education at baseline			19.892	< 0.001
High school diploma	18 (10)	15 (39)		
Some college	65 (38)	13 (33)		
4-year college degree	37 (22)	4 (10)		
Graduate education	52 (30)	7 (18)		

T-tests and exact logistic regressions revealed that mothers who did not smoke during pregnancy were significantly less likely than mothers who smoked to: (1) give verbal dispraises (M 3.14, SD 12.81) (M 22.32, SD 57.55) (t = -2.071, df 38.83, P = 0.022); (2) ever leave bruises (exact OR 8.37, 95% CI 2.46 - 30.83, P < 0.001); (3) verbally reject (exact OR 14.31, 95% CI 1.11 - 769.72, P = 0.039).

A multiple linear regression controlling for demographic differences including total family income, baseline education of the biological mother, ethnicity, and several variables known to impact parenting including alcohol during pregnancy and HBQ-P externalizing scores were controlled and included as covariates shown below. In this analysis, as depicted in Table 2, smoking during pregnancy remained significantly associated with an increase in the frequency of verbal dispraises (P = 0.024).

**Table 2:** Multiple Linear Regression Results Showing Smoking During Pregnancy Status Associated With Frequency of Verbal Dispraises Controlling for Variables Known to Impact Parenting

	Model 1			Model 2^a^		
	
	F (6, 189)	R^2^	P	F (2, 193)	R^2^	P
Overall model	4.74	0.103	< 0.001	12.29	0.104	< 0.001



**Variable**	**Coefficient**	**t**	**P**	**Coefficient**	**t**	**P**

Constant	20.42	2.58	0.011			
Alcohol during pregnancy	-3.55	-1.12	0.264			
Baseline total family income	-0.51	-1.07	0.285			
HBQP externalizing scores	4.66	1.00	0.319			
Race	-2.09	-0.76	0.451			
Education	-1.30	-1.84	0.068	-1.89	-3.60	< 0.001
Smoking during pregnancy	8.91	2.34	0.020	8.42	2.28	0.024

^a^ model 2 used stepwise method to get to the final model.

Smoking during pregnancy was also significantly associated with leaving marks or bruises (intensity) (OR 8.885, 95%CI 2.918, 27.052, P < 0.001) (Table 3). Smoking was not significant for verbal dispraise or verbal rejection (intensity).

**Table 3:** Logistic Regression Results Showing Smoking During Pregnancy Status Associated With Left Marks or Bruises (Intensity) Controlling for Variables Known to Impact Parenting

	**Model 1**				**Model 2^a^**			
	
	**χ^2^**	**df**	**P**	**R^2^**	**χ^2^**	**df**	**P**	**R^2^**
	
Overall model	20.63	5	0.001	0.240	14.69	1	< 0.001	0.173

**Variable**	**Coeff**	**χ^2^**	**P**	**OR^b^** **(95% CI)^c^**	**Coeff**	**χ^2^**	**P**	**OR^b^** **(95% CI)^c^**

Constant	-3.91	11.47	0.001	0.020	-3.25	60.82	< 0.001	0.039
Alcohol during pregnancy	0.27	0.18	0.672	1.30 (0.38, 4.46)					
Baseline total family income	-0.17	3.97	0.046	0.84 (0.71,1.00)					
HBQP externalizing scores	0.79	0.79	0.373	2.20 (0.39, 12.42)					
Education	0.22	2.50	0.114	1.24 (0.95, 1.63)					
Smoking during pregnancy	2.00	9.83	0.002	7.35 (2.11, 25.59)	2.18	14.78	< 0.001	8.89 (2.92, 27.05)

^a^model 2 used stepwise method to get to the final model; ^b^OR: odds ratio; ^c^CI: confidence interval.

## Discussion

The current study findings suggest specific parenting differences in those mothers that smoked during pregnancy compared to those who did not. Pregnancy smokers demonstrated harsher parenting in several specific domains than parents who did not smoke during pregnancy. These differences remained significant even after other factors known to be associated with poor parenting such as low SES, alcohol use, and child’s externalizing behavior scores were accounted for in the analysis. Parenting behaviors among pregnancy smokers remain under-investigated. Current findings suggesting increased levels of harsh parenting in pregnancy smokers extend the extant literature associating pregnancy smoking exposure to child disruptive outcomes. These findings point to modifiable risk factors in pregnancy smokers to ameliorate child disruptive outcomes. While further studies are warranted to elaborate on the mechanisms that drive this association, study results suggest that parenting should be an important target for dyads exposed prenatally to cigarettes. Other studies have suggested treatment intervention to be less effective in children prenatally exposed to cigarettes, warranting further understanding and need for targeted treatment [31]. Regardless of the etiology, targeted psychotherapy addressing these areas appears warranted in this population. As an initial step, psychoeducation could be applied to reduce the use of smoking as a coping mechanism in addition to stress reduction and enhancement of emotion regulation skills in smoking mothers.

Study limitations include a relatively small sample size of pregnancy smokers that were from a longitudinal investigation on depression. Other potential limitations were no bioassay confirmation of smoking measurements and no maternal personality assessment. Additionally, current study findings show less Caucasian and more African American mothers in the group of pregnancy smokers versus non-smokers, making the findings less generalizable to the population. This is also important as multiple smoking investigations find Caucasians to be the ethnic group making up the large majority of pregnancy smokers. Further investigations of such ethnic differences are warranted.

Finally, our reliance on self-reported parenting in the absence of observational parenting measures is a limitation due to the possibility of bias towards appropriate, socially acceptable responses and shared method variance with the same reporter providing information on both predictor and outcome [32]. However, parental self report provides insight into personal subjective experience, and such perceptions are linked to actual parenting behavior and child developmental outcomes [33-35]. The differential association of specific facets of problematic parenting to maternal smoking status in the present study also lends validity to this approach. Future studies which incorporate a multi-method approach to assessment of a broad range of parenting including positive parenting behaviors will help identify critical high impact intervention targets.

### Conclusion

The present investigation compared parenting practices in preschoolers whose mothers reported smoking during pregnancy versus those who did not. Current study findings suggest that smoking during pregnancy is associated with harsh parenting practices. While the current investigation is preliminary and limited by a small sample size, study findings inform the need for larger investigations that specifically examine parenting of younger children among pregnancy smokers, taking into account key variables such as maternal personality characteristics and other related substance use. Future investigations could inform how to improve parenting skills to minimize disruptive behaviors in young children of pregnancy smokers at greatest risk for harsh parenting. Such studies would help identify those most likely to benefit from targeted parenting interventions to optimize public health resources.
